# Prey preference and host suitability of the predatory and parasitoid carabid beetle, Lebia grandis, for several species of Leptinotarsa beetles

**DOI:** 10.1673/1536-2442(2006)6[1:PPAHSO]2.0.CO;2

**Published:** 2006-06-16

**Authors:** Donald C. Weber, Daniel L. Rowley, Matthew H. Greenstone, Michael M. Athanas

**Affiliations:** Insect Biocontrol Laboratory, USDA ARS PSI, BARC-West, Bldg. 011A, Rm. 214, Beltsville, MD 20705

**Keywords:** Colorado potato beetle, biological control, host specificity, food choice

## Abstract

Lebia grandis (Coleoptera: Carabidae), recorded as a parasitoid only on Colorado potato beetle, Leptinotarsa decemlineata (Coleoptera: Chrysomelidae), is capable of parasitizing the false potato beetle, L. juncta, and also L. haldemani. Historical records show that L. decemlineata, while the only recorded host, was not present in much of the original range of L. grandis, and may not have been its host prior to its expansion into eastern North America, where L. juncta is endemic. Our laboratory comparisons suggest that L. juncta, the presumptive original host, best supports the development of the parasitoid larval L. grandis, based on 43.6% successful emergence of the adult carabid parasitoid, compared to 11.5% from the two other Leptinotarsa species. L. grandis adults accept eggs and larvae of all 3 Leptinotarsa species as adult food. Naive, newly-emerged adults show no preference when presented the 3 species of third-instar larvae, which they consume at a mean rate of 3.3 per day, a rate which does not differ significantly by sex, larval host, or weight at emergence. When presented with equal amounts by weight of the 3 species of Leptinotarsa eggs, such adults consume the equivalent of 23.0 L. decemlineata eggs per day, with consumption of L. juncta eggs 67% higher by weight than L. decemlineata consumption. Insight into the biotic and abiotic limitations on L. grandis should aid in determining its potential for suppression of Colorado potato beetle by biological control in diverse agroecosystems.

## Introduction

**Figure 1.**
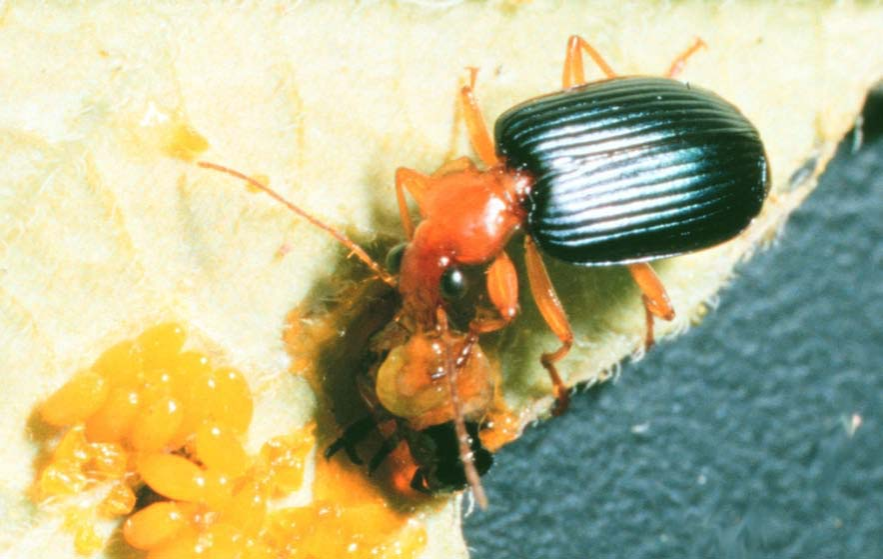
Adult Lebia grandis eating 3rd instar of Leptinotarsa decemlineata.

**Figure 2.**
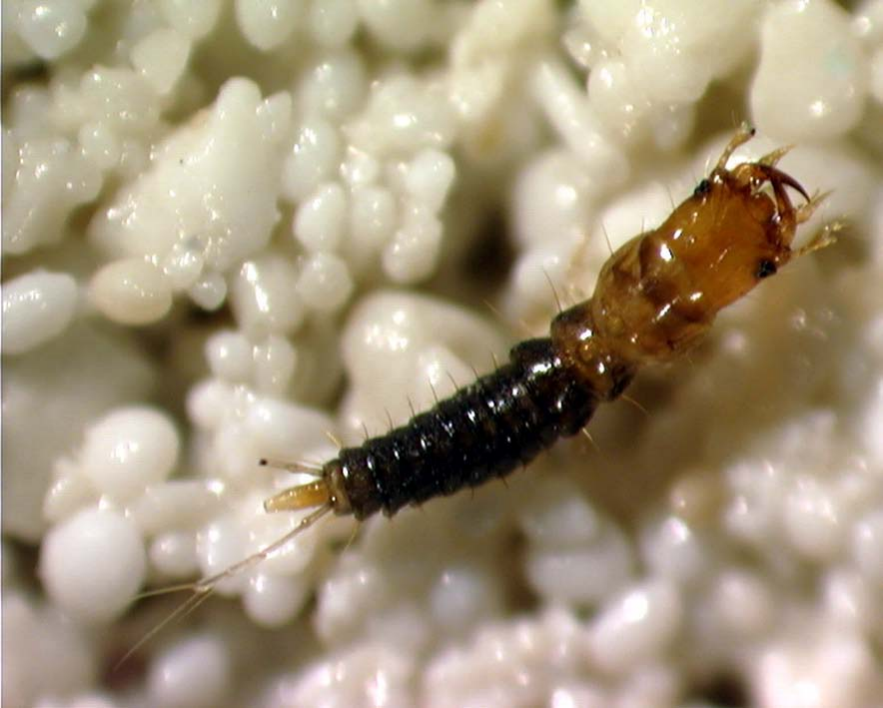
Lebia grandis first instar, prior to any host-feeding, reared at mean temperature of 27.3°C, pictured on tropical sand background.

**Figure 3.**
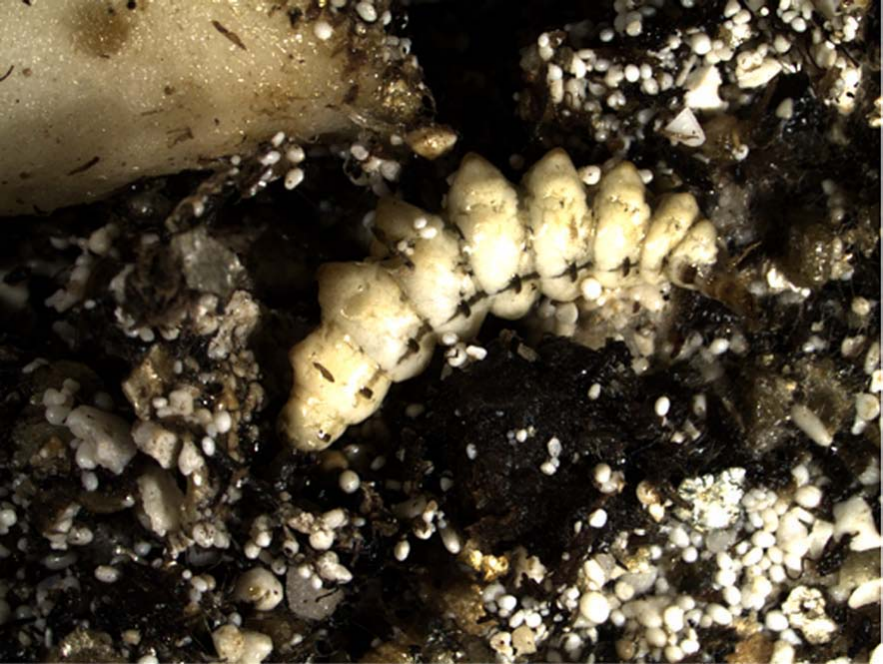
Lebia grandis fed first instar, reared on L. decemlineata in mixed medium and at 27.3°C mean temperature as described in text.

**Figure 4.**
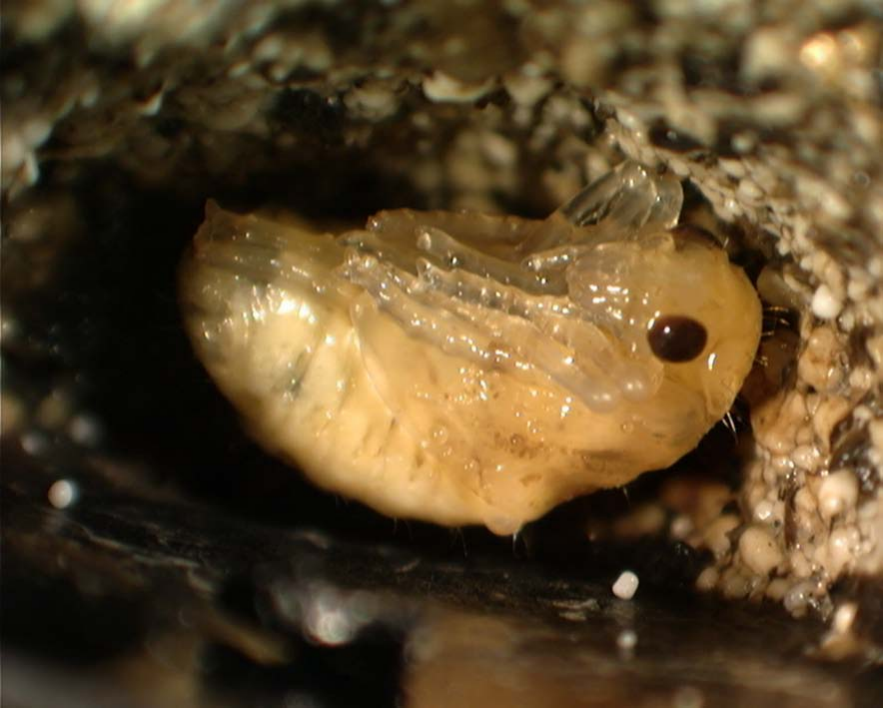
Lebia grandis pupa.

The Colorado potato beetle, Leptinotarsa decemlineata (Say) (Coleoptera: Chrysomelidae), is a major pest of potato, tomato and eggplant in North America and Eurasia. Its resistance to many pesticides (Bishop & Grafius 1996) has prompted development of still more chemical controls, and also management alternatives, including cultural controls, resistant varieties, and endemic and exotic biological controls (Weber 2003). The carabid beetle Lebia grandis Hentz (Coleoptera: Carabidae) is a natural enemy native to North America, whose adults feed on L. decemlineata eggs and larvae; its first-instar larvae are obligate parasitoids of L. decemlineata pupae (Figures 1–4). Lebia grandis is one of the most important endemic predators of L. decemlineata (Groden 1989, Riddick 1999). However, its primarily nocturnal habit, rare appearance in pitfall traps, escape habits of adults, and cryptic life cycle of larvae, all have tended to mask its significance and to result in limited research (Groden 1989).

L. grandis occurs commonly in association with L. decemlineata in the eastern USA as far north as Maine, Ontario and Quebec (Madge 1967; Hemenway & Whitcomb 1967; Lindroth 1969; A. Alyokhin personal communication). But L. grandis historical records (Madge 1967; Lindroth 1969) do not include a large part of the western range of L. decemlineata (Riley et al. 2003). The type locality of North Carolina (Hentz 1830) and several other early records could not have been associated with L. decemlineata, because they were collected before this pest's spectacular and well-documented eastward expansion in the two decades following its adoption of potato as a new host plant, first reported in eastern Nebraska in 1859 (Riley 1876; Tower 1906; Casagrande 1985). L. juncta Germar, the only congener native to the eastern US, is the most likely original host for L. grandis in that region (Lindroth 1969, Riddick 1999). Known as the false potato beetle, this insect feeds on horsenettle (Solanum carolinense L.), and is only occasionally seen on potato (Jacques 1988; Clark et al. 2004). L. haldemani is recorded from Texas, Oklahoma, and Arizona, and many states of Mexico, on wild species of Physalis and Solanum douglasii, and occasionally occurs on potatoes (Jacques 1988; Clark et al. 2004). It may overlap with L. grandis geographically in Texas, but the carabid is not recorded further west or south, nor is there any record of association with L. haldemani (Madge 1967; Lindroth 1969).

A hundred years after its description as a species, L. grandis was still not known to be a parasitoid, and was noted only as a specialist predator of L. decemlineata in the adult stage. This attracted the interest of European researchers after the establishment of L. decemlineata in France in 1922 (Trouvelot 1931). Based on L. grandis adult collections from Long Island, NY, in the 1930s, Chaboussou (1939) established that the larvae had a parasitoid habit, and developed effective rearing conditions in France in furtherance of an unsuccessful attempt at classical biological control of L. decemlineata in France. The first demonstration of the parasitoid life history in a carabid was the congener L. scapularis on elm leaf beetle, Xanthogaleruca luteola (Silvestri 1904). Lebia is one of the few parasitoid genera associated with hosts that feed on living plants (Eggleton & Belshaw 1993), as far as is known exclusively chrysomelids (Riddick 1999). Typically in coleopteran parasitoids, including Lebia, larvae are the host-finding stage (Erwin 1979; Eggleton & Belshaw 1993). Lebia adults are typically found in close association with their host species, and females oviposit in close proximity to the host pupal habitat; in the case of L. grandis, the soil below infested host plants.

There have been few attempts to use predator-parasitoid beetles in pest control. Although the elm leaf beetle is a serious pest on several continents, and classical biological control has been undertaken in the USA and in Australia (Dreistadt & Dahlsten 1990; LeFoe 2002), there is no record of L. scapularis being considered for introductions. However, there has been a large research effort to evaluate and manage several species of the staphylinid genus Aleochara, which parasitize the puparia of cabbage root maggots (Delia radicum (L.) and related species of anthomyiid pests of crucifers) in North America and Europe (Fournet et al. 2000; Soroka et al. 2002). These studies are very promising for use of native and/or introduced species of Aleochara in canola, cole crops, and other crucifers.

Groden (1989) demonstrated that adult L. grandis consume more L. decemlineata eggs or larvae per day than any other L. decemlineata predator. Over the life of the beetle this should have a larger impact than the single prepupa killed by the parasitoid larval stage. However, the “double pest control” (Fournet et al. 2000) offered by the parasitoid-predator life cycle is unique and complex. To gain some initial insight into the biotic potential and limitations of L. grandis, in this study we investigate the host specificity of L. grandis to prey on and to parasitize Leptinotarsa species other than L. decemlineata. First-instar larvae were offered prepupal hosts under no-choice conditions, and newly-emerged adult carabids were offered choices of either three species of Leptinotarsa eggs, or three species of larvae, to determine their breadth and frequency of host and prey acceptance.

## Materials and Methods

### Sources and rearing of chrysomelids and carabids

Lebia grandis adults were collected in August 2003 and July 2004 from potato fields on the USDA Beltsville Agricultural Research Center in Beltsville, Maryland, which had moderate to high populations of L. decemlineata. Groups of eight adults each (of undetermined sex) were housed in 2-liter ventilated plastic containers (18 × 13 × 10cm deep) in growth chambers under a 16:8 L:D photoperiod with relative humidity 50 ± 10% and temperatures of 25°C for the 8-h scotophase ramped over 5 h to 30°C held for 6 h in the middle of the photophase, and ramped down the final 5 h of photophase, for a daily mean temperature of 27.3°C. All experiments described took place under this photoperiod, temperature and humidity regime. Each container also housed two 38 mm long, 10 mm diameter cotton wicks (Patterson Dental Supply, www.pattersondental.com) moistened with deionized water, in a 35 mm diameter open Petri dish bottom, and shredded wax paper covering approximately one-third of the container floor. A 60 cm or 100 cm diameter open 10 mm deep Petri dish bottom, filled with moistened soil mix to within 3 mm of the rim, and with an added 10 mm section of 10 mm diameter cotton wick placed vertically, served as an oviposition substrate that was replaced every 2–4 days. The soil in this egg-dish consisted of a 1:1:1 volume mix of Pro-Mix^™^ (Premier Tech Ltd., www.premiertech.com), tropical sand (Southdown Tropical Play Sand, YardRight Select, www.yardright.com) and horticultural-grade vermiculite (PVP Industries, North Bloomfield, OH), moistened with deionized water to approximately 40% water by weight and periodically re-moistened with deionized water. Adult Lebia were provided with 1.5 to 3 third-instar L. decemlineata larvae every 2 days, from the Insect Biocontrol Laboratory colony reared on potato cv. Kennebec.

Egg-dishes were incubated and checked for hatching of first-instar L. grandis starting seven days after placing them into adult cages, until 8 days after removal. First-instar L. grandis were transferred, one parasitoid to one host, into black Ellipso 1-oz., oval, portion cups with clear lid (Newspring Packaging, www.pactiv.com), into which moistened medium had been filled to within about 5mm of the inner rim. Inside the cup, at the side juncture of medium and plastic, a 10 mm section of 10 mm diameter cotton wick was placed vertically to absorb excess moisture. A starter hole (divot) was made in the center of each filled cup and, using a pointed probe, three ventilation holes were punched into each lid. The divot encouraged the prepupa to dig in the center of the cup and thus pupate surrounded by soil medium. To isolate host prepupae, late 4^th^ instar Leptinotarsa larvae were placed in waxed, 8 oz. ice cream cups (Sweetheart Cup Company, www.solocup.com/) with several host plant leaves; prepupae leave the foliage and try to burrow into the bottom of the cup. Prepupae were placed into the divot of the soil mix, and when the prepupa burrowed into the medium (below the surface level), a single first-instar carabid larva (brown and actively moving) was added and the cup capped. If host prepupae failed to burrow into the medium after one hour, a single carabid larva was nevertheless added. Cups were checked every 1—2 d for emergence of the host or parasitoid, and adult parasitoids used for experiments or added to the colony.

**Figure 5.**
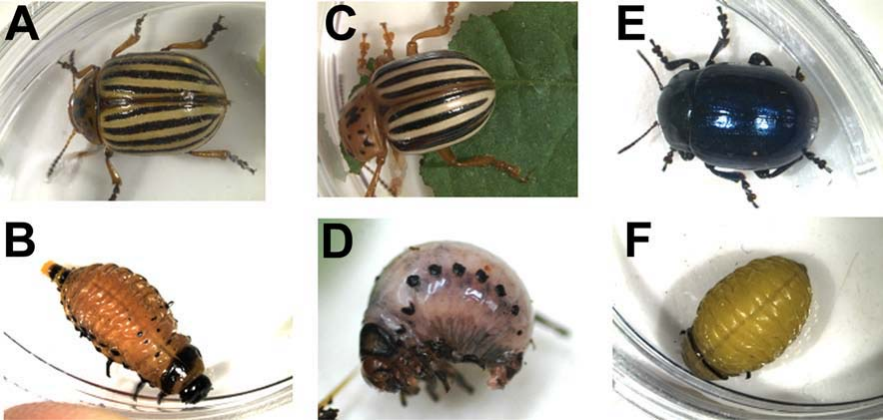
Adults and prepupae of the three Leptinotarsa *species:* 5A, L. decemlineata adult; 5B, L. decemlineata prepupa; 5C, L. juncta adult; 5D, L. juncta prepupa; 5E, L. haldemani adult; 5F, L. haldemani prepupa.

Life stages of L. decemlineata (Figure 5A, 5B) were obtained from Insect Biocontrol Laboratory colonies that originated from Beltsville, Maryland, maintained continuously on a 16:8 L:D photoperiod at room temperature (approx. 25°C), and fed on foliage of potato cv. Kennebec.

L. juncta life stages (Figure 5C, 5D) were collected in 2003 from horsenettle in grazed pastures at the Burgundy Center for Wildlife Studies in Hampshire County, WV, to establish the lab colony for parasitism experiments, and collected as adults in 2004 in potato fields at the USDA Beltsville Agricultural Research Center in Beltsville, MD, to establish the lab colony used for all other experiments. L. juncta are maintained on greenhouse-raised potted horsenettle plants and greenhouse-raised or field-collected horsenettle foliage from Beltsville, MD. Excised foliage is placed in an Aquapic (www.syndicatesales.com) filled with quarter-strength Hoagland's solution (Hoagland and Arnon 1950). Growth chamber rearing conditions are 16:8 L:D photoperiod, temperature of 25°C and relative humidity of 50±10%. Moistened Pro-Mix^™^ is provided for pupation.

L. haldemani adults were collected 15 September 2003 by Richard Crosland from Anderson thornbush (Lycium andersonii Gray) near Benson, AZ. This new host record was established by Gary Bernon and Tom Forrester with a collection in 1986 (G. Bernon, personal communication). L. haldemani (Figure 5E, 5F) were reared on excised potato foliage, without Aquapics, in ventilated plastic 2-liter boxes in growth chambers at 25°C, 50±10% RH and continuous light. Lightly moistened Pro-Mix^™^ is provided for pupation.

### Suitability of Leptinotarsa species for L. grandis parasitism

**Figure 6.**
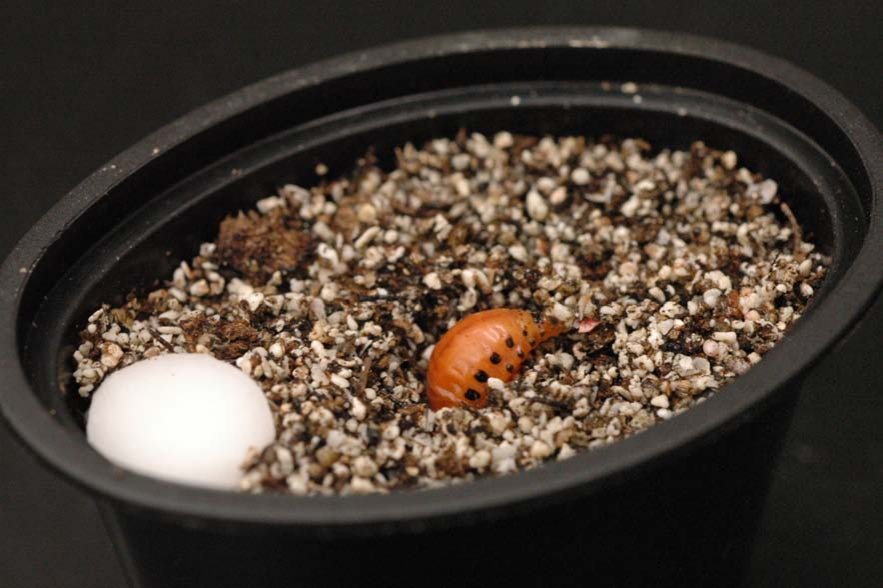
Parasitism setup showing host prepupa (Leptinotarsa decemlineata) before digging.

Host suitability tests were conducted in March and April 2004, using L. grandis first instars and three species of Leptinotarsa prepupae: L. decemlineata, L. juncta, and L. haldemani. L. grandis and Leptinotarsa spp. were obtained from laboratory colonies established the previous summer as detailed above. Host prepupae (4^th^ instar larvae that had ceased feeding) were identified as described above, and weighed to the nearest mg. Prepupae were then set up individually, in 10z cups filled with the above-described mixture of Pro-Mix^™^, sand and vermiculite. The mixture components had been autoclaved for 30 min prior to use and then moistened to 39.0% water content by weight. After weighing, the prepupae were placed into a small divot on the surface of the medium (Figure 6). When the prepupa burrowed into the medium (below the surface level), the elapsed time was recorded and a single first-instar Lebia (fully sclerotized and actively moving) was added and the cup capped. If host prepupae failed to burrow into the medium after 60 min, a Lebia larva was nevertheless added. Half of each host species received one L. grandis larva, and half were allowed to pupate without addition of the larvae, matched so that every second host pupa served as a control. All cups were placed in a growth chamber under a 16:8 L:D photoperiod with relative humidity 50 ± 10% and temperatures of 25°C for the 8-h scotophase ramped over 5 h to 30°C, held for 6 h in the middle of the photophase, and ramped down the final 5 h of photophase, for a daily mean temperature of 27.3°C. Emergence of the host and carabid adults was noted by checking every 1–2 days from 7 until 28 days since installation of the host prepupae. Emerged hosts and parasitoids were weighed to the nearest mg, and sexed.

Chi-square tests (Statview for Windows, SAS Institute 1998) were performed to determine whether the outcomes differed by host. This test was 3 hosts by 2 outcomes (carabid emerged or not emerged from cups to which carabid larvae were added); pairs of hosts were also tested with Bonferroni-corrected 2-by-2 Chi-square tests. The experimental design with matched parasitized and unparasitized treatments allowed a test of whether the introduction of one carabid larva changed the proportion of hosts emerging even in those cups that yielded no carabid adults. This was tested again in a 3 hosts by 2 outcomes (host adult emerged or did not emerge) Chi-square test using only those cups not yielding carabid adults. If host emergence were depressed among those cups not yielding parasitoid adults, this would suggest that a portion of parasitoids caused host mortality, but failed to complete their development, resulting in emergence of neither beetle. Regressions and analysis of covariance (SAS Institute 1998) were used to determine the effect of host mass on emerged parasitoid mass. Whether there was any effect on the sex of the parasitoid arising from either the species or mass of the prepupal host, was determined by comparison with the cumulative binomial distribution, and logistic regression, respectively (SAS Institute 1998). The possible effect on L. grandis adult emergence of time before its prepupal host dug into the soil medium, or whether the host dug at all within 60 min, was tested using logistic regression and Chi-square test respectively. Adult emergence date and mass, both of the parasitoid and its hosts, and as affected by sex of emerged adult, were tested using ANOVA (SAS Institute 1998).

### Larval prey choice of adult L. grandis

Newly-emerged L. grandis adults (the F2 offspring of adults collected in August 2003 from a potato field in Beltsville, Maryland) were weighed within one day of emergence and then offered 2 larvae each of L. decemlineata, L. haldemani, *and* L. juncta, all third instars with an approximate individual weight of 27.0 mg. Thirteen beetles were housed singly in a 300-ml transparent styrene acrylonitrite box (Mepal-Rosti, www.mepal.com) provided with two 23 mm holes covered with gray woven nylon no-see-um cloth (REI Co-op, www.rei.com) secured with hot glue and an opaque white low-density polyethylene snug-fitting top. The container contained two 38 mm long, 10 mm diameter cotton wicks moistened with deionized water in a 35 mm diameter open Petri dish bottom, and shredded wax paper covering one-third of the container floor. The larvae were released in random orientation into a 35 mm open Petri dish top, which was placed in the middle of the container bottom, between the shredded wax paper and the moistened dental wicks. Prey larvae were unfed, and L. grandis adults were released at the beginning of the experiment into the wax paper. At least once each day the larvae were counted and replenished to the original numbers. Following the choice experiment, adult L. grandis were sexed according to the mesotibial character described by Chaboussou (1939). The null hypothesis, that the adults consumed equal numbers of larvae of each species, was tested using Proc Mixed (SAS Institute 2005) to conduct a 2-way ANOVA with one factor indicating whether the distribution is “observed” or “expected” (i.e. equivalent frequency, set to one-third for each species) and the other factor representing the 3 species. Each adult was a replicate experimental unit; the dependent variable was total proportion of prey larvae consumed. Significance of the 2-way interaction effect would indicate non-equivalent preference. The relationship between larval consumption and day was examined by fitting a linear regression using Proc Mixed with TYPE=ANTE(1) covariance structure (among observed consumption amounts for the same adult) specified in the REPEATED statement. The adults arose from a larval rearing using three different larval hosts; this effect as well as sex of Lebia predator was tested as to equal total larval consumption as well.

### Egg prey choice of adult L. grandis

**Figure 7.**
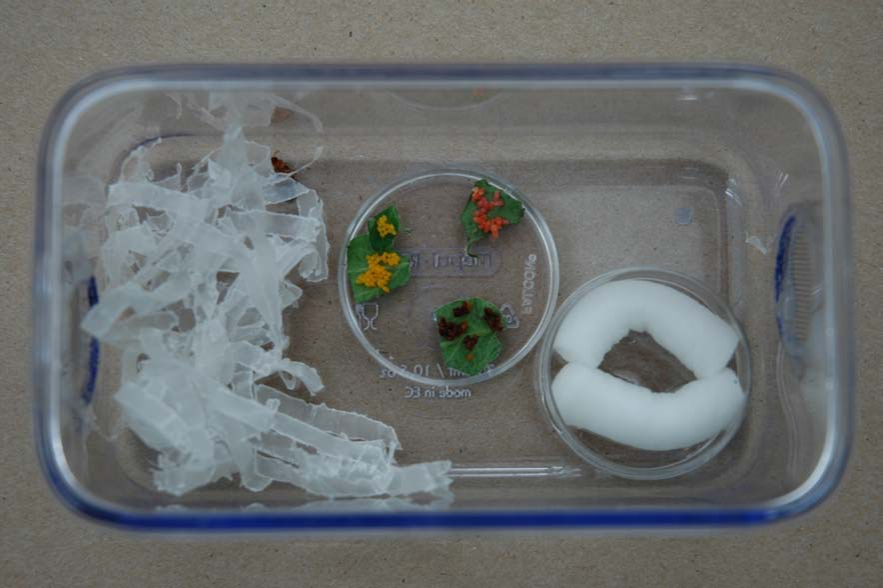
Egg-choice arena showing 3 species of egg masses (each equivalent to 31.4 mg): 21 eggs of Leptinotarsa juncta (pink to salmon in color); 37 eggs of Leptinotarsa haldemani (beige to brown); 50 eggs of Leptinotarsa decemlineata (yellow-orange). These were placed with randomized orientation between moistened cotton wicks and shelter of shredded wax paper in 300 ml container. Note adult Lebia grandis in wax paper.

Twelve newly-emerged L. grandis adults (12 F1 offspring of adults collected in July 2004 from a potato field in Beltsville, Maryland) were weighed within one day of emergence and then housed singly in 300 ml containers as above, and offered 50 eggs of L. decemlineata, 37 eggs of L. haldemani, and 21 eggs of L. juncta, each having an approximate total mass of 31.4 mg. The three egg groups were symmetrically spaced around the margin of a 35 mm open Petri dish top, which was placed in the middle of the container bottom, between the shredded wax paper and the moistened dental wicks, with random egg species orientation (Figure 7). Each day for 5 to 6 days, the eggs were counted and replenished to the original numbers, and any hatching or near-to-hatching eggs also replaced. Following the choice experiment, adult L. grandis were sexed. The null hypothesis, that the adults consumed equal mass of eggs of each species, was tested using Proc Mixed (SAS Institute 2005) to conduct a 2-way ANOVA with one factor indicating whether the distribution is “observed” or “expected” (i.e. equivalent proportion, set to one-third for each species) and the other factor representing the 3 species. Each adult was a replicate experimental unit; the dependent variable was the by-mass proportion of total mass of eggs consumed. Significance of the 2-way interaction effect would indicate non-equivalent preference. The relationship between egg consumption and day was examined as above for larvae.

## Results and Discussion

**Figure 8.**
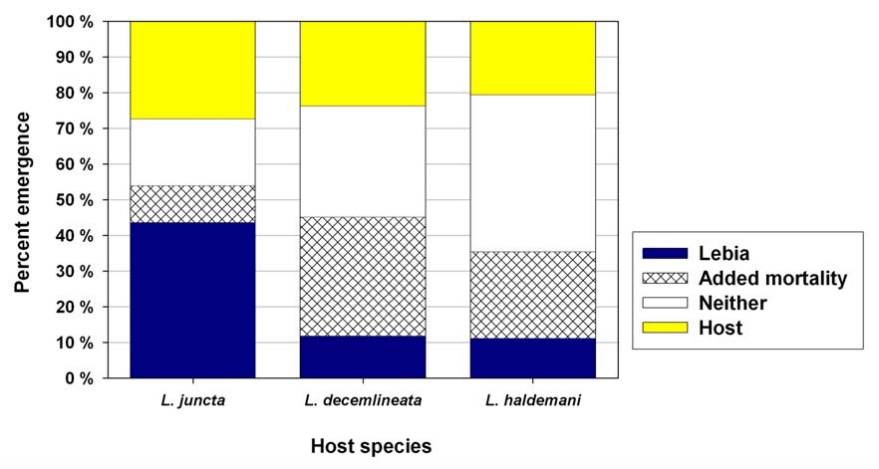
Emergence of the adult parasitoid Lebia grandis, and host beetles, when reared individually on three species of Leptinotarsa. Difference is highly significant for proportions on Leptinotarsa juncta compared to the two other species (p < 10^−6^, χ^2^ test; n = 55,114, and 107 respectively). Crosshatched portion of “neither” denotes added host mortality attributed to parasitoid effect without parasitoid emergence (see text for explanation).

All three Leptinotarsa species supported successful development of L. grandis from first-instar to adult. However, the proportion successfully completing this development differed significantly by host, with 43.6% successful emergence on L. juncta, compared to 11.8% for L. decemlineata and 11.3% for L. haldemani (p<10^−6^; χ^2^ = 30.7, df=2). The proportion of host-parasitoid combinations resulting in no emergence also differed by species (Figure 8). This comparison revealed that for L. decemlineata and L. haldemani, a significant additional mortality associated with the parasitoid occurred in the “Lebia added” treatment, as shown by the crosshatched portion of bars in Figure 8. This proportion was highest for L. decemlineata (33.3%) (2 × 2 test, p<10^−4^; χ^2^ = 29.9, df = 1, overall n = 210), significantly higher than for L. haldemani (24.3%)(p<10^−4^; χ^2^ = 15.8, df = 1, n = 198), and only 10.3% for L. juncta (p = 0.088; χ^2^ = 2.9, df = 1, n = 94; not statistically significantly different from zero but shown in bar graph).

**Figure 9.**
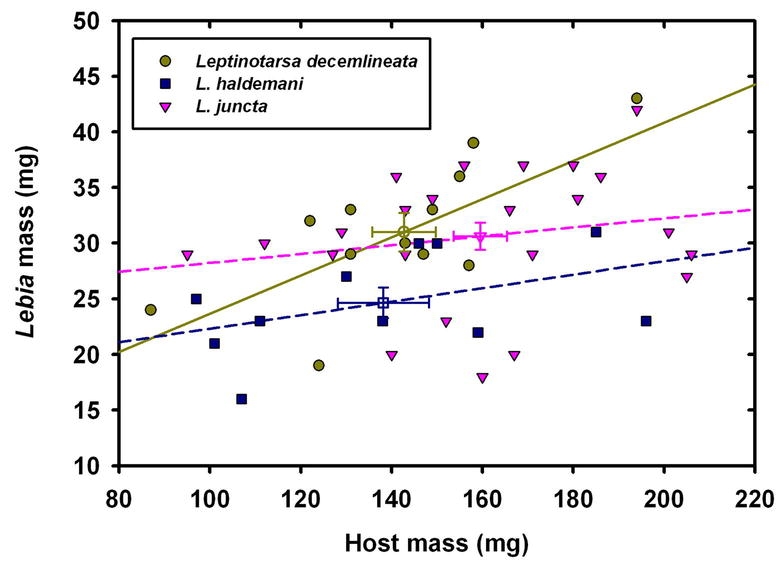
Relationship of Lebia grandis mass (newly-emerged carabid adult) versus host mass (Leptinotarsa prepupa). Slope of regression line is significantly different from zero only for Leptinotarsa decemlineata (solid yellow line: y = 6.51 + 0.172X (n = 13, p= 0.008, R^2^_adj_ = 0.44)); however, slopes for the three hosts do not differ; the significant effect of host is attributable to a reduced mean Lebia mass for those parasitizing Leptinotarsa haldemani, compared to the other two species. Means and standard errors are shown for each host.

The mean adult mass of newly emerged L. grandis was positively related to the prepupal mass of the host, an effect significant only for L. decemlineata (Figure 9 and caption). There was a significant host effect attributable to whether the host was L. haldemani, which depressed adult parasitoid mass by 6.2 mg independent of host mass (Figure 9). There was no interaction of host type and prepupal mass effects, therefore, the regression lines shown are not significantly non-parallel. Means for adult mass of L. grandis based on host were 31.0 mg for L. decemlineata host, 30.6 mg for L. juncta, and 24.6 mg for L. haldemani. Newly emerged male and female adult L. grandis did not differ by fresh mass. Initial mass of the first-instar carabids was not measured, so this parameter could not be examined for influence on the adult emergence mass.

The sex ratio was not significantly different by host nor did this ratio differ significantly from 1:1 for any of the hosts, although there were a total of only 35.9% females emerged and determined. Although host Leptinotarsa differed in mean developmental time from prepupal burying to adult emergence (9.54 days ± 0.14 SE for L. decemlineata, 11.23 ± 0.15 for L. haldemani, and 12.40 days ± 0.16 for L. juncta; all significantly different from one another by Tukey's HSD test at p < 0.05 with an overall F = 90.0 df = 2,60 and p < 0.0001), L. grandis time to emergence did not differ by host, averaging 22.73 days ± 0.45 SE overall. Male and female hosts, and male and female L. grandis, did not differ in days to emergence. This is consistent with the temperature-based model of Groden (1989), which predicts a developmental period of 23.0 days at a constant 27.3°C, and the rearing data of Chaboussou (1939), who reported duration of 20 to 24 days at 25°C.

Although prepupal digging indicates readiness to pupate in Leptinotarsa, and thus might well indicate suitability for L. grandis parasitism, there was no significant effect of time to digging (up to 60 min), or whether the prepupa dug at all within 60 min, on likelihood of successful parasitoid emergence (logistic regression with p = 0.05, R^2^ = 0.026, χ^2^ = 3.9, n = 144; 2 × 2 χ^2^ with p = 0.22, χ^2^ = 1.5, n = 272, respectively, for all hosts pooled). This probably indicates that the prepupae, regardless of immediate readiness to dig, were suitable hosts within the window of first-instar parasitoid activity. According to Groden (1989), the first-instars survived a mean of 4.07 days in two types of moist soil at 25°C. Chaboussou (1939) emphasized that larvae will often fail to parasitize hosts that have already formed their pupation chamber. Thus the risk of asynchrony, at least with young and vigorous parasitoid larvae, may be less with “underripe” prepupae in a one-to-one laboratory setting. There were significant differences among all 3 hosts in proportion digging within 60 min (χ^2^ test, overall p < 0.0001), with 73.5% of L. decemlineata digging, 44.1% of L. juncta, and only 33.3% of L. haldemani.

Under these laboratory conditions, L. decemlineata yielded fewer parasitoids than those used by Chaboussou (1939), who eventually achieved approximately 70% success in cotton-stoppered glass test tube of 1 cm diameter and 12 cm depth, filled with sterilized soil. He emphasized the importance of soil moisture to the success of rearing, desiccation being a hazard early in larval life, and too much moisture inhibiting the pupal-adult molt. It is possible that our containers were too shallow, being only about as deep as the field depth of L. decemlineata pupae in Groden's (1989) tethering experiments, about 3 cm.

Newly-emerged adults presented a choice of 6 third-instar Leptinotarsa larvae, 2 each of the 3 different species, consumed a mean of 3.30 larvae per day (approx. 89.1 mg/d, 55.0% of food offered), with no significant preference among prey species (F = 0.20, p = 0.656). Preference and overall consumption did not differ significantly by predator larval host, sex, emergence mass, or days since emergence (linear or quadratic).

Egg consumption showed a different pattern than larval choice, indicating a difference in egg consumption by species (overall F = 5.32, p = 0.029). The expected proportion of eggs consumed, of the 3 species that were presented in equal masses of 31.4 mg each, was one-third for each species. L. juncta egg consumption (41.4 ± 3.1% of egg fresh mass consumed) was significantly higher than the null hypothesis, whereas L. decemlineata eggs consumed accounted for significantly less (24.7 ± 3.1% of the total), and L. haldemani at 33.9% did not deviate from the expectation under the null hypothesis. Egg consumption averaged 14.4 mg/d (equivalent to the mass of 23.0 L. decemlineata eggs, or 15.3% of total eggs offered) over the 6 days since emergence, and there was no significant linear or quadratic time trend.

Our larval consumption data were higher than those observed by Groden (1989) for field-collected adults held in the lab at 25°C for five days, which consumed 2.52 third-instar L. decemlineata larvae per day. However, our egg consumption was less than Groden's (1989) tests, which show for the same pool of field-collected adults 47.38 L. decemlineata eggs per day at 25°C, and more than 40 per day for 20°C and 30°C as well. Groden's egg consumption results varied over the season, and as Chaboussou (1939) notes, prey consumption varies strongly (from about 0.5 to 5 third instars per day) with both temperature and adult maturity, with an especially marked increase in consumption of L. decemlineata third-instar-larvae (under no-choice conditions) when the females first initiated oviposition. In these experiments, newly eclosed L. grandis ate an estimated mean of 89.1mg/d from Leptinotarsa larval choice, but only a mean of 14.4 mg/d from Leptinotarsa egg choice. The young predators may have been stimulated by the movement of the larvae that wander around the container because they were not fed, promoting repeated predator-prey encounters. Eggs in contrast are stationary, and encounters with these prey require exploration initiated by the predators outside of the shredded wax paper shelter area of the cage. Although our experiments did not test it, newly eclosed L. grandis may simply prefer larvae to eggs. Groden (1989) offered egg-larval choice with the result of no preference; these were field-collected insects of undetermined age, most of which were reproductively mature.

Larval hosts of only four Lebia species have been documented (Silvestri 1904; Chaboussou 1939; Lindroth 1954; Capogreco 1989); ours is the first documentation of multiple hosts for the genus, although such associations have long been suspected (e.g., Capogreco 1989). It is likely, given the large number of observations of Lebia species associated with chrysomelids in the field, that many species have multiple hosts. The carabid parasitoid genus Brachinus parasitizes four beetle families: Hydrophilidae, Dytiscidae, Gyrinidae, and Carabidae (Erwin 1979; Juliano 1985; Saska & Honek 2004). The same parasitoid species may even develop on more than one family of hosts, differing greatly in size. Similar to Lebia, Brachinus larvae are short-lived and their mobility is limited by dispersal ability; this limits host choice, and gives advantage to the ability to develop on a variety of host sizes and types (Juliano 1985). In B. lateralis, larger body size correlates positively with fecundity in females and with mating success in males, and for this species a limited number of data points show a strong relationship of parasitoid mass with host mass, and a suggestion of a different ratio for different hosts (Juliano 1985).

We have demonstrated both quantitative and qualitative host effects on L. grandis for the 3 Leptinotarsa species in this study. Lindroth (1969) remarks that in Lebia, “body-size is remarkably inconstant in many species” due to varying size of the host pupa. Developmental plasticity that allows maturation of smaller-sized adults serves to promote survival under adverse conditions, perhaps also increasing the probability of initial success on novel hosts. This could then be followed by subsequent selection for potentially more efficient utilization of the novel host.

If L. grandis was originally a parasitoid exclusively of L. juncta, its encounter less than 150 years ago with L. decemlineata, a smaller host, represented an enormous opportunity to exploit a novel, extremely abundant food source. Its poorer parasitoid success under our conditions, and lower consumption of L. decemlineata eggs compared to those of L. juncta, suggests that L. decemlineata may still constitute a less-than-optimal host on an individual basis. It is perhaps significant for the parasitoid that L. decemlineata pupae develop almost 3 days more quickly than those of L. juncta (under our lab conditions), possibly detracting from host quality and/or time window for successful parasitoid feeding and development during host metamorphosis. Our study did not explore the possible tritrophic effects of food plant on the carabid parasitoid or predator stages. L. juncta larvae reared on horsenettle (rather than potato, which the other two species were reared on) may be more suitable to Lebia parasitism or predation because of the difference in host plant. Since both L. decemlineata and L. juncta develop well on both eggplant and horsenettle (Weber & Ferro 1996; Weber unpublished for L. juncta on eggplant), such a comparison would elucidate this potential tritrophic effect.

While female body size is frequently positively correlated with fecundity in parasitoids and predators (Jervis & Copland 1996), smaller body size is not always associated with lower fecundity: body size can be completely unrelated to fecundity, since many other factors may be important, such as age at mating and first reproduction, longevity and adult feeding (Leather 1988). For L. grandis, a relatively long-lived, voraciously-feeding adult producing large numbers of very small eggs, body size may not be as important as successfully maturing to a fertile adult within a broad range of body size.

Over the life of the beetle, its predation should have a much larger impact than the single prepupa killed by the parasitoid larval stage. However, the importance of larval specificity to Leptinotarsa is that reproducing adult Lebia are constrained to the habitat of their chrysomelid larval host to a greater extent than any of the other L. decemlineata predators. Thus tethered in time and space to its host, L. grandis has the potential to exert significant biological control on its host over a range of host densities, if environmental conditions are favorable, including chemical and cultural practices in the agroecosystem.

In spite of generalist predators being well-represented in the suite of natural enemies of L. decemlineata in North America and Europe, and providing some important suppression, L. decemlineata frequently outstrips these controls and severely defoliates potato crops in many regions (Hough-Goldstein et al. 1993). Predation of L. decemlineata by generalist heteroptera as well as coccinelids can be disrupted in the presence of alternate prey; this as well as intraguild predation may limit the effectiveness of generalists (Koss & Snyder 2004). The response of the common generalist coccinelid Coleomegilla maculata Lengi to alternate prey is variable and complex (Mallampalli et al. 2005). Therefore, the more specialized Lebia grandis and the tachinid parasitoids Myiopharus spp. (López et al. 1997) may be key species that are disproportionately important (Straub & Snyder 2006) to target for conservation and/or augmentation as part of a strategy to make the potato agroecosystem less favorable for Colorado potato beetle.
